# Epidemiology and risk factors of rectal colonization of carbapenemase-producing *Enterobacteriaceae* among high-risk patients from ICU and HSCT wards in a university hospital

**DOI:** 10.1186/s13756-020-00816-4

**Published:** 2020-09-23

**Authors:** Li Yan, Jide Sun, Xiuyu Xu, Shifeng Huang

**Affiliations:** Department of Laboratory Medicine, the First Affiliated Hospital of Chongqing Medical University, No.1 Friendship Road, Yuzhong District, Chongqing, 400016 China

**Keywords:** Fecal carriage, CRE, CPE, KPC, Risk factors

## Abstract

**Background:**

Nosocomial carbapenemase-producing *Enterobacterieceae* (CPE) infections constitute a major global health concern and are associated with increased morbidity and mortality. Rectal colonization with CPE is a risk factor for bacterial translocation leading to subsequent endogenous CPE infections. This prospective observational study was aimed to investigate the prevalence and epidemiology of rectal colonization of CPE, the carbapenemase genotypes, and to identify the independent risk factors for the acquisition of CPE colonization in high-risk patients from ICU and HSCT wards in a university hospital in China.

**Methods:**

In a prospective cohort study, 150 fecal samples from rectal swabs were consecutively obtained for inpatients from the intensive care unit (ICU) and hematopoietic stem cell transplantation (HSCT) wards from November 2018 to May 2019, and screening test for CPE was conducted by using prepared in-house trypsin soybean broth (TSB) selective media and MacConkey agar. Antimicrobial susceptibility was determined by the broth microdilution method and carbapenemase genes were characterized by both the GeneXpert Carba-R and PCR for *bla*_KPC_, *bla*_NDM_, *bla*_IMP_, *bla*_VIM_ and *bla*_OXA_. Multi-locus sequence typing (MLST) was employed to characterize the genetic relationships among the carbapenemase-producing *K. Pneumonia* (CPKP) isolates. In order to further investigate the risk factors and clinical outcomes of CPE colonization, a prospective case-control study was also performed.

**Results:**

Twenty-six suspected CPE strains, including 17 *Klebsiella pneumoniae*, 6 *Escherichia coli*, 1 *Citrobacter freundii*, 1 *Enterobacter Kobe*, and 1 *Raoultella ornithinolytica,* were identified in 25 non-duplicated rectal swab samples from 25 patients, with a carriage rate of 16.67% (25/150). Through GeneXpert Carba-R and subsequent PCR and sequencing, all the suspected CPE isolates were identified to be positive for the carbapenemase genes, of which 17 were *bla*_KPC_-carriers, and another 9 were *bla*_NDM_-producers. MLST designated all the CPKP isolates to be ST11 clone. Multivariate analysis indicated that urinary system diseases, operation of bronchoscopy, and combined use of antibiotics were independent risk factors for acquiring CPE colonization in high-risk patients from the ICU and HSCT wards.

**Conclusions:**

This study revealed a high prevalence of rectal CPE colonization in high-risk patients from ICU and HSCT wards, and a predominant colonization of the KPC-producing *K. pneumoniae* clone ST11. Stricter infection control measures are urgently needed to limit the dissemination of CPE strains, especially in patients who were afflicted by urinary system diseases, have underwent bronchoscopy, and were previously exposed to combined antibiotic use.

## Background

Carbapenem-resistant *Enterobacteriaceae* (CRE) is a serious public health threat worldwide [1, 2]. CRE infections have been emerging rapidly and posing serious challenges to the clinical management [3], thus, timely and efficient diagnosis, strict and evidence-based infection control measures, and prompt and effective therapy are of paramount importance. To help direct research and development efforts toward the production of novel drugs, CRE was recently listed as one of the three critical-priority pathogens by the World Health Organization (WHO) [4, 5].

Resistance to carbapenems in *Enterobacteriaceae* occurs via one or a combination of carbapenemase production, production of ESBLs and/or AmpC in combination with porin loss/deficiency, carbapenem efflux, or mutations in penicillin-binding proteins (PBPs) [6, 7], among which carbapenemase gene acquisition is of greatest concern [8], as it most frequently occurs via transfer of plasmids, which usually co-harbor β-lactamases and other resistance determinants, rendering these carbapenemase-producing *Enterobacteriaceae* (CPE) strains multi-drug resistant (MDR) or extensively drug-resistant (XDR) [9].

CPE is the most problematic due to higher-level antimicrobial resistance, clonal spread and horizontal gene transfer [10]. Carbapenemases include Ambler class A β-lactamases (e.g., KPC and GES), class B metallo-β-lactamases (MBLs, e.g., NDM, VIM, IMP, and SPM), and class D β-lactamases (e.g., OXA-48 and OXA-181) [6]. In the case of CPE, only some “second-line” drugs, such as polymyxins, tigecycline, and fosfomycin, and some “last resort” antibiotics, such as aztreonam/avibactam [11], ceftazidime/avibactam [12], meropenem/vaborbactam [13], and imipenem/relebactam [14] have been demonstrated to be effective in some cases. In addition, although several newer investigational agents that have not trialed against CPE yet, such as eravacycline [15], plazomicin [16], and cefiderocol [17], may be effective against CPE, but is still speculative. Also, resistance to these new drugs can be expected to emerge, as has been seen with aztreonam/avibactam [18] and ceftazidime-avibactam [19]. It is therefore crucial to implement efficient infection control measures to limit the spread of these pathogens.

The lack of control measures directed at CPE carriers has led to the spread of CPE in healthcare settings. Screening for asymptomatic CPE colonization and implementing contact precautions will reduce patient-to-patient transmission. Although evidence-based guidelines on prevention and control of CPE are available [20–22], CPE still remains as a severe challenge in health care settings, and more studies on appropriate countermeasures are required especially for high-risk patients from intensive care unit (ICU) and hematopoietic stem cell transplantation (HSCT) wards. This prospective observational study was aimed to investigate the prevalence and epidemiology of fecal carriage of CPE, the carbapenemase genotypes, and to identify the independent risk factors for the acquisition of CPE colonization in high-risk patients in ICU and HSCT wards from a university hospital in China.

## Methods

### Setting and study design

The present study was a single-center prospective survey performed at the first Affiliated Hospital of Chongqing Medical University, China. Our hospital is a 3200-bed teaching hospital providing all types of acute care with around 200,000 admissions and 1000,000 hospital-days per year.

We implemented a prospective analysis of a cohort of CPE-colonized patients detected from November 2018 to May 2019. All hospitalized patients in ICU wards (including central ICU, respiratory ICU, critical surgery ICU, neurology ICU, neurosurgery ICU, thoracic and cardiac surgery ICU) and HSCT wards during this period were included, due to the high prevalence of CRE carriage observed in these wards in the baseline survey, and are thus considered the highest-risk sites for CRE transmission.

Rectal swabs were obtained from all patients in a representative sample of wards in ICU and HSCT. Each cohort patient was systematically screened for CPE colonization on the day of admission. A total of 150 rectal swabs were collected from non-repetitive patients. Stool samples collected from rectal swab were cultured on selective TSB media (Pangtong, China), and the suspected CPE isolates were further identified at the species level by the VITEK MS (bioMérieux, MO, USA) system, and routine antimicrobial susceptibility testing was performed using the VITEK2 compact (bioMérieux, Inc., NC, USA) system.

### Bacterial identification and antimicrobial susceptibility testing

A total of 26 clinical CRE strains were isolated and identified from November 2018 to May 2019 by using the VITEK MS (bioMerieux, Hazelwood, MO, United States) automated system at the department of laboratory medicine, all of which were resistant to at least one carbapenem on the basis of antimicrobial susceptibility testing results determined by the broth microdilution method, with the criteria of MIC of ≥2 mg/L for ertapenem, ≥ 4 mg/L for imipenem, and ≥ 4 mg/L for meropenem.

### Detection of antibiotic resistance genes

Both GeneXpert Carba-R (*bla*_KPC_, *bla*_NDM_, *bla*_IMP_, *bla*_VIM_ and *bla*_OXA_) and PCR were used to confirm the suspected resistance phenotypes. Moreover, the Carba NP test and sCIM were performed on all isolates to determine whether any bacteria produced carbapenemases by phenotypic methods but were negative by genotypic methods, or vice versa.

Multi-locus Sequence Typing (MLST) of the 17 *bla*_KPC_-carrying *K. pneumoniae* isolates.

In order to further characterize the genetic relationships among the 17 *bla*_KPC_-carrying carbapenemase-producing *K. Pneumonia* (CPKP) isolates, multi-locus sequence typing (MLST) was performed by the amplification and subsequent sequencing of the internal fragments of seven housekeeping genes of *K. Pneumonia* isolates (*rpoB*, *gapA*, *mdh*, *pgi*, *phoE*, *infB*, and *tonB*) and comparing them with KP MLST database (https://bigsdb.pasteur.fr/klebsiella/klebsiella.html) to determine the allele types and STs of CPKP isolates.

### Risk factors for CPE colonization

We conducted a prospective case-control study to explore the risk factors and clinical outcomes of patients colonized with CPE from November 2018 to June 2019 in Chongqing, China. Patients with CPE colonization were included as cases. Controls were identified as patients without CPE colonization with well-balanced demographic characteristics, pre-existing medical conditions, and immune-compromising comorbidities as compared with the cases. Clinical and epidemiological data, including the demographics, underlying diseases, the primary diagnosis at admission, invasive procedures prior to the isolation of CPE, previous exposures of antibiotic within 3 months, and the clinical outcomes, were extracted from the patients’ electronic medical records system and clinical microbiology laboratory database.

### Statistical analysis

Data were collected using Excel software (Microsoft, Redmond, CA, USA), and were described using the mean with standard deviation (SD) for continuous variables and proportions (%) for qualitative variables.

All analyses were performed using SPSS v.22.0 software (SPSS Inc., Chicago, IL, United States). Univariate analyses were performed separately for each of the variables. Categorical variables were compared using a chi-square test or Fisher’s exact test as appropriate. Continuous variables were compared using Student’s t-test (normally distributed variables) and Wilcoxon rank-sum test (non-normally distributed variables) as appropriate. The odds ratio (OR) and 95% confidence interval (CI) were calculated to evaluate the strength of any association. Variables with *P* ≤ 0.05 on univariate analysis were evaluated as potential covariates in a stepwise multivariate logistic regression model. All *P*-values were two sided, and *P* < 0.05 was considered significant.

### Ethical considerations

The data and samples analyzed in the present study were obtained in accordance with the standards and approved by the Chongqing Medical University Institutional Review Board and Biomedical Ethics Committee.

## Results

### Prevalence of positive CRE colonization among high-risk patients from ICU and HSCT wards

In the CRE prevalence survey, 150 patients from ICU and HSCT wards were prospectively recruited, and a total of 150 non-repetitive fecal swabs were collected, among which 26 CRE strains from 25 individual patients were detected as being CRE positive, with a carriage rate of 16.67% (25/150). Among the 26 CRE isolates, 22 CRE isolates (84.62%, 22/26) were isolated from 21 individual patients in the ICU wards, with a carriage rate of 18.58% (21/113); another 4 were from 4 patients in the HSCT wards, with a carriage rate of 10.81% (4/37). The 26 isolated CRE strains included 17 *Klebsiella pneumoniae* (65.38%), 6 *Escherichia coli* (23.08%), 1 *Citrobacter freundii* (3.85%), 1 *Enterobacter Kobe* (3.85%), and 1 *Raoultella ornithinolytica* (3.85%)*.* The 26 CRE strains were isolated from 25 non-duplicated samples from 25 individual patients (14 male and 11 female; 21 from ICU and 4 from HSCT) whose mean age was 63.48 ± 22.81 years. Among these patients, 40.0% (10/25) were transferred from another hospital.

### Confirmation of carbapenemase-producing phenotypes and molecular characterization of carbapenemase genes

Both Carba NP test and sCIM were performed on all isolates to determine whether the CRE isolates produced carbapenemases by phenotypic methods. Among the 26 CRE isolates, all were positive in both the two carbapenemase phenotype confirmatory tests, in concordance with our later molecular characterization results from both GeneXpert Carba-R and subsequent PCR and sequencing, which demonstrated 26 CPE isolates with carbapenemase genes. As was shown in Table 1, the most prevalent carbapenemase gene was *bla*_KPC_ (65.38%, 17/26), detected in all the 17 *K. pneumoniae* isolates; followed by *bla*_NDM_ (34.62%, 9/26), detected in all of the 6 *Escherichia coli* isolates, 1 *Citrobacter freundii*, 1 *Enterobacter Kobe*, and 1 *Raoultella ornithinolytica* isolate; *bla*_IMP_, *bla*_VIM_, and *bla*_OXA-48_ were not detected (Table 1). Notably, all the 17 *K. pneumoniae* isolates harbored *bla*_KPC_, while all the 6 carbapenemase-producing *Escherichia coli* strains carried *bla*_NDM_. It’s also worth noting that one patient was demonstrated to co-harbor two CPE colonization isolates, with one *K. pneumoniae* strain producing KPC, and another *E. coli* isolate expressing NDM. Moreover, all the 26 CPE isolates showed resistance to all the three tested carbapenems (imipenem, meropenem, and ertapenem), showing a perfect correlation between CPE phenotype and genotype.

**Table 1:** Molecular characterization of carbapenemase genes in 26 CRE isolates as detected by GeneXpert Carba-R and PCR

Bacteria	Carbapenemases (isolate number)				
	***bla***_**KPC**_	***bla***_**NDM**_	***bla***_**VIM**_	***bla***_**IMP**_	***bla***_**OXA-48**_
*Klebsiella pneumoniae*	17	–	–	–	–
*Escherichia coli*	–	6	–	–	–
*Citrobacter freundii*	–	1	–	–	–
*Enterobacter Kobe*	–	1	–	–	–
*Raoultella ornithinolytica*	–	1	–	–	–

### MLST typing results

To further characterize the genetic relationships among the 17 *bla*_KPC_-carrying CPKP isolates, MLST was carried out. All the 17 *bla*_KPC_-carrying CPKP isolates were exclusively assigned to ST11, clearly demonstrating that resistance is restricted to one genetic background and that it is a problem of inter-hospital spread of one clone.

### Risk factors for the acquisition of rectal CPE colonization in high-risk patients from ICU and HSCT wards

The risk factors for the acquisition of rectal CPE colonization in high-risk patients from ICU and HSCT wards were shown in Table 2. The univariate analysis indicated that higher APACHE II Scores, diabetes mellitus, urinary system disease, coronary heart disease, endocrine system diseases, multiple organ dysfunction syndrome, bronchoscopy, and combined use of antibiotics were significantly more frequent in patients with rectal CPE colonization (*P* < = 0.05). Further multivariate logistic regression analysis demonstrated that urinary system disease (OR [Odd ratio]: 18.06, 95% CI [Confidence Interval]: 3.31–98.62, *p* = 0.001), operation of bronchoscopy (OR: 4.05, 95% CI: 1.30–12.60, *p* = 0.01), and previous exposure to combined antibiotics (OR: 3.60, 95% CI: 1.18–10.93, *p* = 0.02) were independent risk factors for the acquisition of rectal CPE colonization in high-risk patients from ICU and HSCT wards.

**Table 2:** Univariate and multivariate analyses of risk factors for patients with CPE colonization

Variable	CRE Colonization (***n*** = 25)	Control (***n*** = 75)	Univariate analysis		Multivariate analysis	
			OR (95% CI)	***P***-value	OR (95% CI)	***P***-value
**Patient characteristics**						
Man gender (Male)	14 (56.00%)	48 (64.00%)	0.65 (0.21–2.00)	0.45		
Age	63.48 ± 22.81	60.76 ± 19.01	1.01 (0.98–1.05)	0.36		
Transferring from another hospital	10 (40.00%)	37 (49.33%)	0.92 (0.29–2.92)	0.88		
**Underlying diseases**						
PITT Scores	2.28 ± 1.86	2.98 ± 2.09	0.79 (0.54–1.15)	0.22		
APACHE II Scores	17.16 ± 7.18	15.74 ± 8.13	1.10 (1.01–1.20)	**0.05**	0.40 (0.09–1.71)	0.22
Hypertension	13 (52.00%)	38 (50.66%)	0.62 (0.18–2.15)	0.42		
Diabetes	12 (48.00%)	20 (26.67%)	3.97 (1.16–13.62)	**0.03**	4.30 (0.67–27.306)	0.12
Tuberculosis	2 (8.00%)	4 (5.33%)	0.94 (0.10–8.50)	0.95		
Hepatitis B	2 (8.00%)	3 (4.00%)	2.17 (0.24–19.57)	0.09		
Tumour	12 (48.00%)	21 (28.00%)	2.88 (0.85–9.72)	0.09		
Respiratory diseases	17 (68.00%)	36 (48.00%)	2.64 (0.76–9.14)	0.13		
COPD	9 (36.00%)	5 (6.67%)	0.92 (0.20–4.20)	0.91		
Hepatobiliary disease	8 (32.00%)	26 (34.67%)	0.63 (0.18–2.10)	0.47		
Gastrointestinal disease	6 (24.00%)	27 (36.00%)	0.36 (0.09–1.46)	0.15		
Chronic kidney disease	6 (24.00%)	21 (28.00%)	0.75 (0.21–2.70)	0.66		
Urinary system disease	10 (40.00%)	6 (8.00%)	7.71 (2.21–26.83)	**0.001**	18.06 (3.31–98.62)	**0.001**
Cardiovascular disease	15 (60.00%)	41 (54.67%)	0.87 (0.27–2.80)	0.82		
Coronary heart disease	7 (28.00%)	10 (13.33%)	4.26 (1.12–16.28)	**0.03**	3.29 (0.91–11.98)	0.07
Neurological disease	9 (36.00%)	18 (24.00%)	2.27 (0.64–7.99)	0.20		
Disease of immune system	1 (4.00%)	5 (6.67%)	0.22 (0.02–2.85)	0.25		
Hematological system diseases	4 (16.00%)	16 (21.33%)	1.47 (0.32–6.72)	0.62		
Endocrine System Diseases	15 (60.00%)	28 (37.33%)	3.16 (1.05–9.54)	**0.04**	1.62 (0.33–7.99)	0.55
Surgery in the past 6 months	2 (8.00%)	16 (21.33%)	0.32 (0.05–1.99)	0.22		
Multiple organ dysfunction syndrome	6 (24.00%)	8 (10.67%)	6.27 (1.35–29.05)	**0.02**		
Transplant	4 (16.00%)	11 (14.67%)	1.82 (0.43–7.64)	0.42		
Urinary tract infection	10 (40.00%)	12 (16.00%)	3.64 (0.78–16.87)	0.09		
Respiratory infection	21 (84.00%)	51 (68.00%)	2.20 (0.37–12.92)	0.38		
Abdominal infection	8 (32.00%)	2 (2.67%)	0.48 (0.05–4.68)	0.53		
Septic shock	1 (4.00%)	9 (12.00%)	0.99 (0.95–1.02)	0.53		
Severe anemia	5 (20.00%)	17 (26.15%)	1.06 (0.27–4.13)	0.94		
Glucocorticoid	7 (28.00%)	12 (16.00%)	2.58 (0.86–9.72)	0.21		
Cardiotonic	2 (8.00%)	5 (6.67%)	0.78 (0.08–7.87)	0.84		
Receipt of total parenteral Nutrition (days)	39.00 ± 57.47	16.71 ± 25.33	0.99 (0.95–1.02)	0.53		
Urinary catheter (days)	56.08 ± 60.35	21.80 ± 26.07	1.04 (0.99–1.09)	0.08		
Nasal catheter (days)	35.38 ± 48.75	21.59 ± 36.98	0.97 (0.92–1.04)	0.16		
Mechanical ventilation (days)	34.64 ± 48.61	15.15 ± 19.01	1.02 (0.98–1.09)	0.16		
Trachea cannula	24.80 ± 48.02	12.65 ± 24.27	0.98 (0.95–1.03)	0.55		
Tracheotomy	10 (40.00%)	31 (41.33%)	0.17 (0.02–1.28)	0.08		
Bronchoscopy	12 (48.00%)	13 (17.33%)	8.15 (1.61–41.22)	**0.01**	4.05 (1.30–12.60)	**0.01**
Drainage tube	19 (76.00%)	55 (73.33%)	0.31 (0.05–1.76)	0.18		
Penicillin	5 (20.00%)	17 (22.67%)	0.60 (0.14–2.55)	0.48		
Cephalosporins	8 (32.00%)	25 (33.33%)	0.37 (0.08–1.61)	0.19		
Carbapenem	10 (40.00%)	24 (32.00%)	1.12 (0.32–3.84)	0.86		
Fluoroquinolone	5 (20.00%)	11 (14.67%)	0.87 (018–4.30)	0.87		
Tetracycline	3 (12.00%)	4 (5.33%)	0.93 (0.14–5.90)	0.92		
Glycopeptide	3 (12.00%)	12 (16.00%)	0.20 (0.03–1.15)	0.07		
Tigecycline	4 (16.00%)	3 (4.00%)	1.94 (0.27–13.98)	0.51		
Combined use of antibiotics	18 (72.00%)	32 (42.67%)	5.91 (1.20–29.16)	**0.03**	3.60 (1.18–10.93)	**0.02**

## Discussion

CPE transmission has been enabled by the lack of control measures directed at CPE carriers in healthcare settings. Screening patients for asymptomatic colonization and implementation of contact precautions could reduce patient-to-patient transmission [23]. Investigation of the prevalence of the rectal CPE colonization among high-risk patients from ICU and HSCT wards can contribute to better infection control measures to limit CPE dissemination. Our study revealed that the rate of rectal CPE colonization in the high-risk patients from ICU and HSCT wards was 16.67%, with the sole carbapenemase gene being *bla*_KPC_ in *K. pneumoniae* isolates and *bla*_NDM_ in *E. coli*. Stricter infection control measures are urgently needed to limit the CPE dissemination, especially in patients who were afflicted by the urinary system diseases, have underwent bronchoscopy, and were previously exposed to combined antibiotic use.

While CRE infection rates were reported to be varied (from 7.6 to 44.4%) in individual studies for CRE-colonized patients [24, 25], a recent meta-analysis suggested an overall 16.5% risk of infection with CRE amongst patients colonized with CRE [26]. From a clinical point of view, all patients in whom colonization could represent a risk factor for invasive disease should be screened [27]. Notably, chemotherapy for acute leukemia, solid organ transplantation, and ICU stay were identified as the most important patient-related conditions associated with a significant risk of CRE infections in colonized patients [28]. Accordingly, with this indication, ICUs, Transplant Units, Hematological Units, major surgical and Infectious Disease Units represent the preferential setting for active targeting screening. We thus focused on the investigation of the prevalence of CPE colonization in patients from ICU and HSCT wards in our hospital. As expected, this study revealed a higher rate of fecal carriage of CPE (16.67%) in the high-risk patients from ICU and HSCT wards than that was reported for randomly recruited patients from several previous studies in Fujian (6.6%) [29], Hunan (8.5%) [30], and Thailand (8.7%) [31].

Rapid detection and characterization of carbapenemase types in colonized CPE strains according to the Ambler classification will lead to improved guidance on the implementation of infection control measures. In this study, we have demonstrated that carbapenem resistance in rectal *K. pneumoniae* and *E. coli* isolates was respectively solely associated with KPC and NDM production. On the other hand, as selective pressure from carbapenem use continues, multiple carbapenemases per pathogen, including pathogens carrying three different carbapenemase genes [32, 33], or multiple CPE pathogens per patient are increasingly common. In our screening, although non CPE isolates co-harboring double or triple carbapenemases were identified, we witnessed one patient co-colonized by two CPE isolates, with one KPC-producing *K. pneumoniae* strain and another NDM-expressing *E. coli* strain. As these deleterious pathogens may spread throughout health care facilities, closer attention to infection control measures and stewardship of the carbapenem-containing drugs should be paid in order to control selection of even more detrimental pathogens.

CPKP strains are the most clinically prominent CPE. Ever since the first report of CPKP in 2001 from the United States [34], an increase in CPKP isolates, largely attributed to ST11, ST15, ST101, ST258, and ST512 strains, was reported across Europe [35]. CPKP isolates emerging from the Indian Ocean rim [36], the United States [37], and China [38] have further exacerbated the global burden of CPKP. The genetic relationship among the 17 KPC-producing CPKP clones was tracked by MLST. Surprisingly, all the 17 *bla*_KPC_-carrying CPKP isolates were exclusively designated as Clone ST11, indicating that carbapenem-resistance in *K. pneumoniae* is limited to clone ST11. Although the dissemination mechanism is still unclear, our findings highlighted the importance of long-term active resistance surveillance of CPKP in high-risk patients from both ICU and HSCT wards.

High prevalence of CPE carriers may cause difficulties for patient flow, as well as high costs due to the need for contact isolation. In order to prevent colonization, the investigation of the clinical predictors and risk factors for rectal CPE colonization admits of no delay. A recently published case-control study demonstrated solid organ and stem cell transplantation, mechanical ventilation, fecal incontinence, and exposure in the prior 30 days to carbapenems, vancomycin, and metronidazole as independent factors associated with CRE colonization [39]. In this prospective case-control study, operation of bronchoscopy, urinary system diseases, and combined use of antibiotics were demonstrated to be independently associated with rectal CPE colonization in high-risk patients from the ICU and HSCT wards. One possible explanation for bronchoscopy operation as an independent risk factor may be due to the fact that invasive bronchoscopy operation might have damaged the patients’ natural immune barriers, and possibly led to CPE colonization. Given that invasive procedures such as the use of urinary catheters were frequently performed in patients with urinary system diseases, it is also easy to understand urinary system diseases as another independent risk factor for rectal CPE colonization. As invasive procedures were potential risk factors, infection control measures preventing the microbial colonization of the insertion sites are necessary. In addition, our study identified combined use of antibiotics as being associated with rectal CPE colonization. One possibility is that inappropriate combined antibiotic use may disrupt the gastrointestinal microflora and eradicate susceptible competing strains, thus elevating the incidence of CPE colonization [40].

Our study had several limitations. Firstly, this was a prospective single-center case-control study with relatively small sample size, and our results might not be applicable to other settings. Secondly, we didn’t investigate the subsequent CPE infection rates in these CPE-colonized patients.

## Conclusion

In conclusion, this study provided a detailed report of the prevalence, molecular epidemiology, and risk factors for rectal CPE colonization in high-risk patients from ICUs and HSCT wards in one medical center. Our data showed a high prevalence of rectal CPE colonization in high-risk patients from ICU and HSCT wards, and a predominant colonization of the ST11-type, KPC-producing *K. pneumoniae* strains. A bundle of infection control and prevention measures with an anti-infective stewardship program is urgently needed to reduce the rectal CPE colonization, especially in patients who were afflicted by urinary system diseases, have underwent bronchoscopy, and were previously exposed to combined antibiotic use.

## Data Availability

All the data of this article is available from the corresponding author if reasonably requested.

## Abbreviations

CLSI: Clinical and Laboratory Standards Institute
CRE: Carbapenem-resistant *Enterobacteriaceae*
CPE: Carbapenemase-producing *Enterobacteriaceae*
MIC: Minimum inhibitory concentration
WHO: World health organization
ETP: Ertapenem
IMP: Imipenem
MEM: Meropenem
